# Anti-Osteoporotic and Anti-Adipogenic Effects of the Water Extract of *Drynaria roosii* Nakaike in Ovariectomized Mice Fed a High-Fat Diet

**DOI:** 10.3390/molecules24173051

**Published:** 2019-08-22

**Authors:** Seon-A Jang, Youn-Hwan Hwang, Taesoo Kim, Ami Lee, Hyunil Ha

**Affiliations:** Herbal Medicine Research Division, Korea Institute of Oriental Medicine, Yuseong-daero 1672, Yuseong-gu, Daejeon 34054, Korea

**Keywords:** *Drynaria roosii* Nakaike, osteoporosis, adipogenesis, menopause, ovariectomy

## Abstract

In traditional oriental medicine, *Drynaria roosii* Nakaike is widely used in treating bone diseases. Postmenopausal women are strongly associated with osteoporosis and obesity. This study aimed to investigate the effects of the water extract of *D. roosii* (WDR) on bone loss and obesity in ovariectomized (OVX) mice fed a high-fat diet (HFD). Body weight, gonadal fat weight, histological findings, and morphometric parameters in trabecular bone were evaluated after OVX mice were treated with WDR and HFD for four weeks. The receptor activator of nuclear κ-B ligand (RANKL)-induced osteoclast differentiation in bone marrow-derived macrophages (BMMs) was examined. Phytochemical identification of WDR using ultrahigh-performance liquid chromatography–tandem mass spectrometry was performed. WDR reversed the changes in body weight gain, gonadal fat mass, and trabecular bone parameters by ovariectomy. However, ovariectomy-induced uterine atrophy was not affected by WDR. WDR decreased adipocyte size and pro-inflammatory cytokines (interleukin (IL)-1β and IL-6) in gonadal fats and lipid accumulation in the bone marrow, which were induced by ovariectomy. WDR significantly decreased RANKL-induced osteoclast differentiation in BMMs. Fifteen phytochemicals were identified in WDR: Seven and nine with anti-osteoporotic and anti-adipogenic activities, respectively. Our findings suggest that WDR may have beneficial effects on postmenopausal osteoporosis and obesity.

## 1. Introduction

Menopause results from a depleted pool of follicles in the gonads and from estrogen deficiency, which leads to physiological alterations, including changes in bone loss and lipid metabolism [1,2]. The normal bone turnover cycle is impaired by estrogen deficiency in postmenopausal women, which increases the prevalence of osteoporosis and fractures. During the menopausal transition period, osteoblastic activity decreases, whereas the osteoclastic resorption activity increases, which leads to a net bone loss. This is due to a weakened inhibitory effect caused by the decrease in useful estrogen on both osteoclast activity and osteoclastogenesis [3]. In addition, estrogen regulates fat distribution and adipocyte differentiation, thus increasing the risk of weight gain and obesity in postmenopausal women. Excessive weight gain and obesity or abnormal fat accumulation are major risk factors of many chronic diseases [4]. Obesity is associated with hypertrophy and hyperplasia of adipocytes and excessive visceral fat tissue growth and affects adipose tissue functionality. Compared with subcutaneous fat, visceral fat is more associated with increased inflammatory responses, producing a higher amount of pro-inflammatory cytokines, including interleukin-6 (IL-6), interleukin-1β (IL-1β), and tumor necrosis factor-α (TNF-α) [5,6]. Moreover, compared with premenopausal women, abdominal fat is deposited more than subcutaneous fat in postmenopausal women due to physiological changes in lipid metabolism [7]. Postmenopausal obesity is related to metabolic pathologies, such as diabetes, non-alcoholic fatty liver, and cardiovascular disease.

*Drynaria roosii* Nakaike belongs to the Polypodiaceae plant family, and its rhizome called Gol-Se-Bo in Korean and Gu-Sui-Bu in Chinese has been used in traditional herbal medicine for treating broken bones. In clinical trials including the rhizome of *D. roosii* in postmenopausal women with osteoporosis, its anti-osteoporotic effect was similar or excellent compared with several osteoporosis treatment agents including estradiol valerate, tibolone, and medroxyprogesterone in terms of bone mineral density without side effects [8,9]. Pharmacological studies have demonstrated the bone-protective effects of *D. roosii* and the underlying mechanisms in both cell culture and animal studies. Lee et al. reported that *D. roosii* enhanced the bone mass of ovariectomized (OVX) rats, and previous studies showed that it improved osteoblast activity and suppressed osteoclast functions [10,11,12,13]. Furthermore, besides the bone-protective effects of *D. roosii*, it has an estrogen-like protective effect, promotes angiogenesis, prevents nephrotoxicity, and enhances kidney function [14,15,16]. Despite various studies showing the beneficial effects of *D. roosii*, its simultaneous modulatory effect on both postmenopausal osteoporosis and obesity has not been studied.

The OVX mice, a useful model for menopausal women, showed increased susceptibility to bone loss, weight gain, and metabolic dysregulation. Administration of high-fat diet (HFD) to OVX mice can further exacerbate weight gain, body fat accumulation, and related inflammation [17,18]. Although studies on the effects of HFD on bone have shown conflicting results, depending on multiple factors, such as gender and age [19], it has been shown that HFD increases body weight but does not affect bone loss in OVX mice [20]. Consistent with previous reports [17,20], body weight gain, gonadal fat weight, and adipocyte size in OVX mice fed HFD (60 kcal% fat) increased more than those in OVX mice fed a low fat diet (10 kcal% fat) or sham-operated mice with HFD, and HFD did not affect OVX-induced bone loss (our unpublished data). In this regard, the present study investigated the effects of the water extract of *D. roosii* (WDR) on bone and fat accumulation in OVX mice fed HFD, an animal model of postmenopausal osteoporosis and obesity.

## 2. Results and Discussion

### 2.1. Effects of WDR on Bone Loss in HFD-Fed OVX Mice

**Figure 1:**
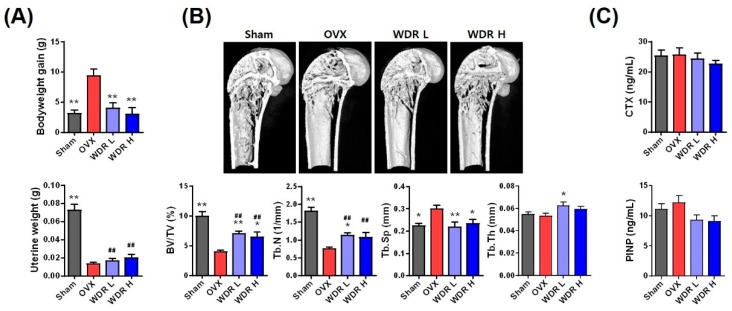
Effects of water extract of *D. roosii* (WDR) on bone loss in high fat diet (HFD)-fed ovariectomized (OVX) mice. (**A**) Changes in body and uterine weight. (**B**) µ-CT images and morphometric parameters in the distal femur. (**C**) Serum levels of CTX-1 and PINP. Sham, sham mice fed HFD OVX, OVX mice fed HFD; WDR L, OVX mice fed HFD and WDR 200 mg/kg/day; WDR H, OVX mice fed HFD and WDR 500 mg/kg/day. Results are presented as mean ± SEM and analyzed using a one-way analysis of variance and Dunnett’s post hoc test. * *p* < 0.05, ** *p* < 0.01 versus the OVX group, ^##^ *p* < 0.01 versus the Sham group.

We first investigated the effect of WDR on body weight gain in OVX mice fed HFD. Figure 1A shows the body and uterine weight of each group. Consistent with previous reports, the bodyweight of the OVX group was markedly higher than that of the Sham group [17,20]. The bodyweight of mice in both WDR L (200 mg/kg/day) and WDR H (500 mg/kg/day) groups were markedly lower than those in the OVX group. The used doses of WDR were chosen based on a previous study that showed significant alleviation of OVX-induced reductions in bone mineral contents after oral administration of WDR at a dose of 500 mg/kg/day to rats [13]. The uterus is one of the most estrogen-responsive reproductive tissues and can easily atrophy with estrogen deficiency. In contrast to weight gain, the uterine weight of the OVX group was dramatically lower than that of the Sham group, which is consistent with previous reports [21]. Treatment with WDR did not affect ovariectomy-induced uterine atrophy, and WDR was presumed to have less estrogenic activity.

Another characteristic of the estrogen-deficient menopausal model is osteoporosis. Menopausal osteoporosis only alters the trabecular architecture unlike senile osteoporosis (due to aging) [22,23]. Morphological examination of bone microstructure under pathophysiological conditions can provide crucial information regarding the degree of bone loss. To estimate the effect of WDR treatment on trabecular bone structure, trabecular architectural parameters were measured in the distal femoral bone by using micro-computed tomography (µ-CT) analysis (Figure 1B). In the OVX group, the bone volume per tissue volume (BV/TV) and trabecular number (Tb.N) were significantly lower, whereas trabecular separation (Tb.Sp) was significantly higher than that in the Sham group. The BV/TV and Tb.N in the WDR groups significantly increased compared with those in the OVX group, whereas Tb.Sp was decreased compared with that in the OVX group. However, trabecular thickness (Tb.Th) in the OVX group did not differ from that in the Sham group, whereas that in the WDR L group was markedly higher than that in the OVX group. The BV/TV ratio is an important parameter in the evaluation of the microstructure of the trabecular bone [24]. A lower BV/TV ratio is associated with fewer trabecular bones and morphological features, such as disconnected and rod-shaped trabecular bones [25]. WDR treatment restored trabecular connectivity by increasing Tb.N and BV/TV ratio and reducing Tb.Sp.

Biochemical markers of bone turnover are produced during the bone remodeling process, which can be detected in the blood and urine. Therefore, the levels of procollagen type 1 N-terminal propeptide (PINP) and C-terminal cross-linked telopeptides of type I collagen (CTX-1), which are used as markers of bone formation and resorption, respectively, were measured (Figure 1C). Unexpectedly, the PINP and CTX-1 levels were not associated with morphometric observations. Collectively, our results demonstrate that WDR alleviates OVX-induced architectural deterioration in trabecular bone independently of estrogenic activity.

### 2.2. Effects of WDR on Osteoclast Differentiation In Vitro

**Figure 2:**
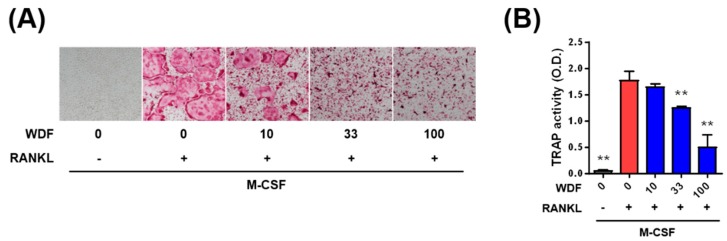
Effects of WDR on osteoclast differentiation in vitro. (**A**) Tartrate-resistant acid phosphatase (TRAP) staining and (**B**) TRAP activity of osteoclasts induced by RANKL in BMMs. BMMs were cultured for four days with or without WDR (10–100 μg/mL) and RANKL (50 ng/mL) in the presence of M-CSF (60 ng/mL). Results are presented as mean ± SEM and analyzed using a one-way analysis of variance and Dunnett’s post hoc test. ** *p* < 0.01 versus the RANKL control group.

Osteoclast differentiation is stimulated by osteoclastogenic factors such as macrophage colony-stimulating factor (M-CSF) and receptor activator of nuclear κ-B ligand (RANKL). M-CSF induces the proliferation and survival of osteoclast precursor cells, and RANKL promotes differentiation of osteoclast precursors into osteoclasts [26]. We investigated whether WDR inhibits osteoclast differentiation stimulated by RANKL in osteoclast precursors, called bone marrow-derived macrophage (BMMs). In the presence of M-CSF, osteoclast differentiation and tartrate-resistant acid phosphatase (TRAP) activity stimulated by RANKL were inhibited by WDR in a dose-dependent manner (Figure 2A,B). In addition, 10–100 μg/mL of WDR did not affect the cell viability of BMMs, presenting that the inhibitory effect of WDR was not due to cell proliferation or cytotoxicity (data not shown). Therefore, these results suggest that the anti-osteoclastogenic effect of WDR might contribute to its inhibitory action on OVX-induced trabecular bone loss.

### 2.3. Effects of WDR on Fat Accumulation in HFD-Fed OVX Mice

**Figure 3:**
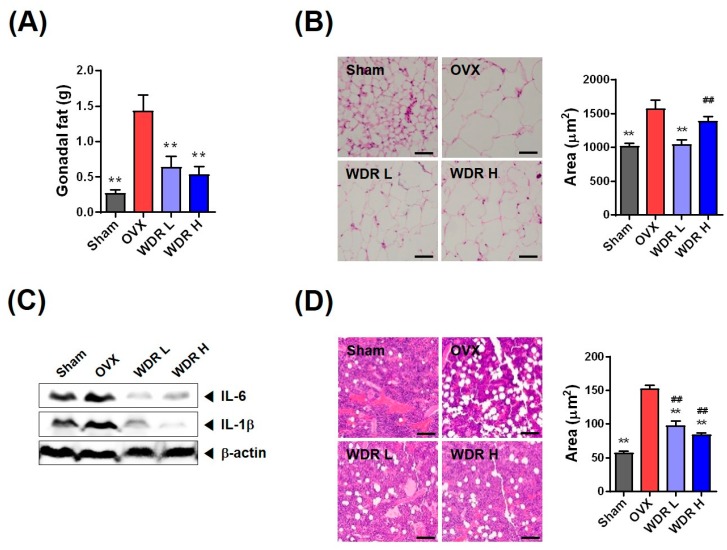
Effects of WDR on fat accumulation in HFD-fed OVX mice. (**A**) Change in gonadal fat weight. (**B**) Hematoxylin and eosin staining of gonadal fat tissue and the mean adipocyte size (×100). (**C**) Protein expression of IL-6 and IL-1β in gonadal fat. (**D**) Hematoxylin and eosin staining of the distal femur and the mean lipid droplets size (×100). Sham, sham mice fed HFD; OVX, OVX mice fed HFD; WDR L, OVX mice fed HFD and WDR 200 mg/kg/day; WDR H, OVX mice fed HFD and WDR 500 mg/kg/day. The results are presented as mean ± SEM and analyzed using a one-way analysis of variance and Dunnett’s post hoc test. ** *p* < 0.01 versus the OVX group, ^##^ *p* < 0.01 versus the Sham group.

Through the menopausal transition in women exhibited by an increase in fat accumulation and change in fat distribution, visceral fat composed of gonadal, retroperitoneal, mesenteric, and perirenal fat are deposited more than subcutaneous fat in postmenopausal women compared with premenopausal women [27,28,29,30]. HFD-fed OVX mice are used as an experimental model of postmenopausal metabolic syndrome due to exacerbation for fat accumulation and related inflammation, and their lipid metabolic profiles are well characterized [17,18]. Figure 3A shows the gonadal fat weight of each group. The gonadal fat weight of the OVX group was markedly increased compared with that of the Sham group, whereas that of the WDR groups was substantially reduced compared with that of the OVX group (Figure 3A). Thus, our results suggest that weight loss in the WDR groups can be attributed to the reduced fat weight.

The increase in gonadal fat mass results from adipocyte hypertrophy and/or hyperplasia, and bigger adipocytes secrete increased amounts of pro-inflammatory cytokines, such as TNF-α, IL-6, and IL-1β, which are involved in lipid metabolism and inflammation [31]. Previous studies have shown that OVX and HFD increased gonadal adipocyte size and elevated levels of inflammatory cytokines [17]. Consistent with these studies, the OVX group had an increased incidence of adipocyte hypertrophy in gonadal fat tissue compared with the Sham group, whereas WDR treatment suppressed adipocyte hypertrophy induced by ovariectomy (Figure 3B). Moreover, the protein expression of IL-6 and IL-1β in gonadal fat tissue was increased in the OVX group compared with the Sham group, whereas increased inflammatory cytokines were significantly decreased by WDR treatment (Figure 3C). Thus, these results suggest that WDR supplementation inhibits inflammatory cytokine expression by decreasing the adipocyte size in HFD-fed OVX mice.

Increased bone marrow adiposity can exacerbate s and inhibit fracture healing and regeneration [32,33]. Fat accumulation in the femoral bone marrow was measured to estimate the effect of WDR treatment on bone marrow adiposity (Figure 3D). Ovariectomy significantly increased fat accumulation in the femoral bone marrow, which was inhibited by WDR treatment. The three common factors of osteoporotic bone in estrogen-deficient postmenopausal women are increased osteoclast formation, increased bone marrow fat production, and decreased osteoblast formation. During osteoporosis transition, bone marrow mesenchymal stem cells (BM-MSCs) show a decreased ability to differentiate into osteoblasts and increased ability to differentiate into adipocytes, which decreases bone formation and increases bone marrow fat accumulation [34,35,36,37]. Thus, WDR treatment is speculated to suppress increased marrow fat accumulation by regulating the shift between osteoblast differentiation and adipocyte differentiation of BM-MSCs. In sum, these findings suggest that WDR may inhibit fat accumulation in the bone marrow and gonadal fat tissue in postmenopausal obesity. However, further studies are required to determine the mechanisms of how WDR reduces obesity and fat accumulation.

### 2.4. Phytochemical Profiles of WDR

**Figure 4:**
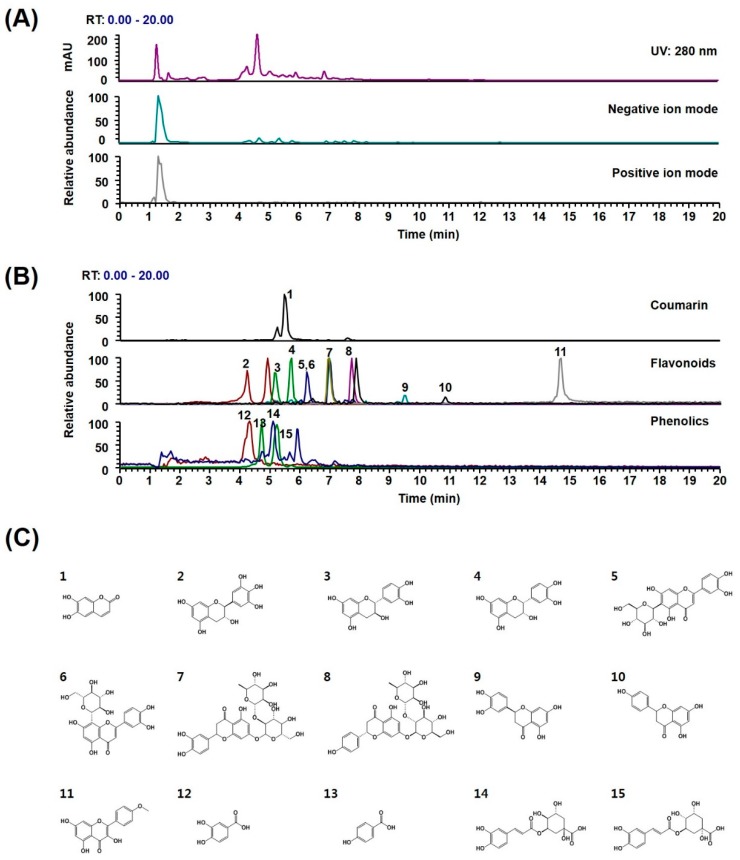
UHPLC–MS/MS analysis of WDR. (**A**) UV and base peak chromatograms of WDR. (**B**) Extracted ion chromatogram of the 15 identified chemical constituents. (**C**) Chemical structures of the constituents. RT, retention time. The peak numbers correspond to the compounds mentioned in (**C**) and Table 1.

Further investigation of phytochemicals in the WDR was performed to expand the information needed to clarify biological properties and potential underlying mechanisms. Currently, *D. roosii* has been reported to contain over 360 flavonoids, phenolic acids, triterpenoids, and lignans [38]. Ultrahigh-performance liquid chromatography–tandem mass spectrometry (UHPLC–MS/MS) analysis of WDR identified one coumarin (esculetin), 10 flavonoids ((−)-gallocatechin, (−)-catechin, (−)-epicatechin, isoorientin, orientin, neoeriocitrin, naringin, eriodictyol, naringenin, and kaempferide), and four phenolics (protocatechuic acid, neochlorogenic acid, p-hydroxybenzoic acid, and chlorogenic acid) by comparing the retention times and mass fragmentations of authentic standards (Figure 4 and Table 1). The UV and base peak chromatograms of WDR are shown in Figure 4A, and the extracted ion chromatograms for each WDR analyte are shown in Figure 4B. The profile of phytochemicals identified in WDR coincides with that in previous reports [10,38,39,40]. The anti-osteoporotic effects of coumarin (esculetin), flavonoids (naringin, eriodictyol, naringenin, and kaempferide), and phenolics (protocatechuic acid and chlorogenic acid) have been reported in in vitro and in vivo studies [41,42,43,44,45,46,47,48,49,50,51,52,53,54,55,56,57]. These phytochemicals inhibited RANKL-induced osteoclastogenesis and osteoclastic bone resorption in vitro and suppressed lipopolysaccharide or ovariectomy-induced bone loss in vivo. The inhibitory effect was associated with the downregulation of the expression of c-Fos and nuclear factor of activated T-cells cytoplasmic 1 (NFATc1), key transcription factors for osteoclast differentiation, and osteoclast specific genes (e.g., DC-STAM, OC-STAMP, Atp6v0d2, Ctr, CtsK, TRAF6, MMP, c-Src, β3-integrin). Besides anti-osteoporotic activities, coumarin (esculetin), flavonoids ((−)-epicatechin, orientin, naringin, naringenin, and kaempferide), phenolics (protocatechuic acid and chlorogenic acid), and medicinal plants containing these constituents reportedly inhibit adipogenesis [58,59,60,61,62,63,64,65,66,67,68,69,70]. These phytochemicals inhibited the expression of adipogenic transcription factors, such as PPARα and C/EBPβ, during adipocyte differentiation of 3T3-L1 cells and BM-MSCs. Therefore, the pharmacological effects of WDR on HFD-fed OVX mice may result from the useful effects of the above-mentioned constituents. However, further studies are necessary to demonstrate the effects and mechanisms of action of the bioactive constituents under HFD-fed OVX conditions.

## 3. Materials and Methods

### 3.1. Reagents and Chemicals

Alpha-modified minimal essential medium (α-MEM), fetal bovine serum (FBS), and phosphate-buffered saline were obtained from Thermo Fisher Scientific Inc. (Rockford, IL, USA). Recombinant M-CSF was provided by Dr. Yongwon Choi (University of Pennsylvania School of Medicine, Philadelphia, PA, USA). Recombinant soluble RANKL was prepared as previously described [71]. Fast red violet, naphthol AS-MX phosphate, and p-nitrophenyl phosphate were purchased from Sigma-Aldrich (St. Louis, MO, USA). Cell Counting Kit-8 was purchased from Dojindo Molecular Technologies Inc. (Rockville, MD, USA).

### 3.2. WDR Preparation

The dried rhizome of *D. roosii* (0.5 kg), which was authenticated by an expert botanist, Dr. J. So (the National Development Institute of Korean Medicine, Gyeongsan, South Korea), was extracted with distilled water at boiling point under reflux for 3 h and lyophilized after filtration. A voucher specimen was stored in the herbarium (registration number #JW-3) of the Korean Institute of Oriental Medicine. The lyophilized powder (WDR) was stored at −20 °C and dissolved in distilled water before use.

### 3.3. Experimental Animal and Diets

Female C57BL/6J mice (six weeks) were purchased from Japan SLC Inc. (Shizuoka, Japan) and acclimated for one week. The mice were provided with water and standard mouse chow ad libitum. They were housed at standard conditions (22 °C ± 2 °C and 55% ± 5% humidity under a 12 h light/dark cycle). All experimental protocols were approved by the Institutional Animal Care and Use Committee at Knotus (Guri, South Korea). The mice were OVX or sham-operated and again acclimated for one week. The mice were randomly divided into four groups (*n* = 5): (1) Sham mice fed HFD (Sham), (2) OVX mice fed HFD (OVX), (3) OVX mice fed HFD and WDR 200 mg/kg/day (WDR L), and (4) OVX mice fed HFD and WDR 500 mg/kg/day (WDR H) groups. The mice had free access to water and commercial HFD (60 kcal%; Research Diet, New Brunswick, NJ, USA), and WDR was administered given by oral gavage once daily for four weeks.

### 3.4. µ-CT Bone Analysis

The distal femur was scanned by µ-CT (PerkinElmer, Inc., Hopkinton, MA, USA) to determine structural changes in bone architecture. Alteration in trabecular bone architecture was evaluated using SkyScan software version 1.4.3.2 (Kontich, Belgium). For trabecular bone analysis of the distal femur, the measurement volume started 100 μm from the lower end of the growth plate, and extended for 150 cross-sections (1.5 mm high). Bone morphometric parameters including BV/TV, Tb. N, Tb. Sp, and Tb.Th were calculated.

### 3.5. Histological Analysis

The femur was collected and fixed using 4% formaldehyde. The tissue samples were then dehydrated, embedded into paraffin, and sectioned at 5-μm thickness. Gonadal fat tissue was cryosectioned at 10-μm thickness. The sections were stained with hematoxylin and eosin and then examined using an optical microscope.

### 3.6. Cell Culture and TRAP Assay

BMMs were cultured in α-MEM medium containing 10% FBS, 1% penicillin/streptomycin, and M-CSF (60 ng/mL). BMMs were treated with or without WDR (10–100 μg/mL) in the presence of M-CSF (60 ng/mL) and RANKL (50 ng/mL) for four days. The cells were fixed using 4% formalin, permeabilized using 0.1% Triton X-100, and incubated with TRAP buffer (50 mM sodium tartrate and 0.12 M sodium acetate, pH 5.2) containing 1 mg/mL of p-nitrophenyl phosphate for 20 min at 37 °C, and the reaction was measured at 405 nm. The cells were also stained for TRAP activity using TRAP buffer with 0.5 mg/mL of fast red violet and 0.1 mg/mL of naphthol AS-MX phosphate.

### 3.7. Western Blot Analysis

Gonadal fat tissues were lysed in radioimmunoprecipitation assay buffer containing the Halt protease and phosphatase inhibitor cocktail (Thermo Scientific, Rockford, IL, USA). The lysates were collected by centrifugation at 13,000× *g* for 30 min at 4 °C. Protein concentration was measured with the BCA protein assay kit (Bio-Rad Laboratories, Hercules, CA, USA). Protein samples were electrophoresed using a 12% sodium dodecyl sulfate-polyacrylamide gel electrophoresis and transferred to polyvinylidene fluoride membrane. The membranes were blocked with 5% nonfat milk and incubated with primary antibody against IL-1β, IL-6, and β-actin overnight. The membranes were washed in tris-buffered saline and tween 20 for 30 min and then incubated with horseradish peroxidase-conjugated secondary antibodies for 1 h. Specific bands were detected using Pierce ECL Western blotting substrate and visualized using the ChemiDoc Touch Imaging System (Bio-Rad).

### 3.8. UHPLC–MS/MS Analysis

Fifteen reference standards were used to identify the chemical constituents of WDR (Table 1). Esculetin, (−)-gallocatechin, (−)-catechin, (−)-epicatechin, isoorientin, orientin, neoeriocitrin, naringin, eriodictyol, naringenin, kaempferide, protocatechuic acid, neochlorogenic acid, p-hydroxybenzoic acid, and chlorogenic acid were purchased from TargetMol (purity, >95%, Boston, MA, USA). Liquid chromatography–mass spectrometry grade formic acid, acetonitrile, and water were purchased from Thermo Fisher Scientific. A Dionex UltiMate 3000 system equipped with Thermo Q-Exactive mass spectrometer was used as described in previously reported methods [72]. WDR and reference standards were prepared in methanol. Xcalibur v.3.0 and Tracefinder v.3.2 software were utilized for data acquisition and analysis.

### 3.9. Statistical Analysis

The results are presented as mean ± SEM and analyzed using a one-way analysis of variance and Dunnett’s post hoc test using Prism version 7. A level of *p* < 0.05 was considered statistically significant.

## 4. Conclusions

In summary, this study showed the dual effects of WDR on postmenopausal osteoporosis and obesity. WDR significantly inhibited RANKL-induced osteoclast differentiation in vitro possibly by suppressing c-Fos and NFATc1, key transcription factors for osteoclast differentiation. In addition, WDR decreased not only bone loss but also fat accumulation in the adipose tissue and bone marrow in HFD-fed OVX mice, indicating that WDR is a potential candidate that can simultaneously manage both osteoporosis and obesity in postmenopausal women. However, the active constituents of WDR and their mechanisms of action in postmenopausal women need to be elucidated further in future studies.

## Figures and Tables

**Table 1:** Profile of chemical constituents of WDR by UHPLC–MS/MS.

No	t_R_ (min)	Precursor Ion (*m*/*z*)			Elemental Composition	Error (ppm)	MS/MS Fragments (*m*/*z*)	Identification	References
		Estimated	Calculated	Adduct					
**Coumarin**									
1	5.5	179.0339	179.0339	[M + H]^+^	C_9_H_6_O_4_	0.169	179, 163	Esculetin *	-
**Flavonoids**									
2	4.2	305.0669	305.0667	[M − H]^−^	C_15_H_14_O_7_	0.803	305, 219	(−)-Gallocatechin *	[39]
3	5.1	289.0721	289.0718	[M − H]^−^	C_15_H_14_O_6_	1.327	289, 245, 109	(−)-Catechin *	-
4	5.7	289.0721	289.0718	[M − H]^−^	C_15_H_14_O_6_	1.221	289, 245, 203, 125	(−)-Epicatechin *	[38]
5	6.2	447.0938	447.0933	[M − H]^−^	C_21_H_20_O_11_	1.188	447, 327	Isoorientin *	-
6	6.2	447.0938	447.0933	[M − H]^−^	C_21_H_20_O_11_	1.188	447, 357, 327	Orientin *	-
7	6.9	595.1676	595.1668	[M − H]^−^	C_27_H_32_O_15_	1.281	595, 459, 151	Neoeriocitrin	[40]
8	7.7	579.1725	579.1719	[M − H]^−^	C_27_H_32_O_14_	0.959	459, 271	Naringin *	[40]
9	9.4	287.0565	287.0561	[M − H]^−^	C_15_H_12_O_6_	1.423	287, 151, 135	Eriodictyol *	[40]
10	10.8	271.0615	271.0612	[M − H]^−^	C_15_H_12_O_5_	1.193	271, 151	Naringenin	[10]
11	14.7	299.0563	299.0561	[M − H]^−^	C_16_H_12_O_6_	0.754	299, 284	Kaempferide *	-
**Phenolics**									
12	4.3	155.034	155.0339	[M + H]^+^	C_7_H_6_O_4_	0.589	132, 114, 155	Protocatechuic acid *	[38]
13	4.7	353.0882	353.0878	[M − H]^−^	C_16_H_18_O_9_	1.006	353, 191, 179, 135	Neochlorogenic acid *	-
14	5.1	139.0391	139.0390	[M + H]^+^	C_7_H_6_O_3_	0.665	139, 121	P-hydroxybenzoic acid *	-
15	5.2	353.0881	353.0878	[M − H]^−^	C_16_H_18_O_9_	0.920	191, 179, 135	Chlorogenic acid *	[39]

* Compared with the retention time (tR) and MS spectral data of authentic standards.

## Abbreviations

WDR	water extract of *D. roosii*
OVX	ovariectomized
HFD	high-fat diet
M-CSF	macrophage colony-stimulating factor
RANKL	receptor activator of nuclear κ-B ligand
BMM	bone marrow-derived macrophage
